# Semen Quality and Metabolic Biomarker Changes Following a 12-Week Combined Exercise Program in Healthy Adult Men: A Non-Randomized Controlled Intervention Study

**DOI:** 10.3390/jcm15218415

**Published:** 2026-10-29

**Authors:** Ahmet Emin Doğan, Barış Sarıakçalı, Nuriye Sarıakçalı, Heidar Sajedi, Mehmet Bayansalduz, Yunus Şahinler, Levent Ceylan

**Affiliations:** 1 Department of Urology, Ankara Etlik City Hospital, 06170 Ankara, Türkiye; ahmetemindogan@gmail.com; 2 Department of Internal Medicine, Faculty of Medicine, Sivas Cumhuriyet University, 58140 Sivas, Türkiye; drbbarissariakcali@gmail.com; 3 Department of Nursing, Faculty of Health Sciences, Sivas Cumhuriyet University, 58140 Sivas, Türkiye; nsariakcali@cumhuriyet.edu.tr; 4 Department of Exercise and Sports Science for Disabled People, Faculty of Health Sciences, International Science and Technology University, 02-264 Warsaw, Poland; heidar.sajedi@istu.edu.pl; 5 Coaching and Movement Sciences Department, Faculty of Sport Sciences, Düzce University, 81620 Düzce, Türkiye; 6 Department of Sports Management, Faculty of Sport Sciences, Istanbul Topkapi University, 34330 Istanbul, Türkiye; mehmetbayansalduz@topkapi.edu.tr; 7 Department of Exercise and Sport Sciences for Persons with Disabilities, Faculty of Sport Sciences, Istanbul Gelisim University, 34310 Istanbul, Türkiye; ysahinler@gelisim.edu.tr; 8 Department of Sports Management, Faculty of Sport Sciences, Hitit University, 19030 Çorum, Türkiye

**Keywords:** concurrent training, male reproductive health, sperm motility, glucose metabolism, insulin resistance, testosterone, sex hormone-binding globulin, lifestyle intervention

## Abstract

**Background/Objectives:** Male reproductive health is increasingly considered an integrated physiological outcome influenced by lifestyle, body composition, metabolic status, and endocrine regulation. This study examined changes in semen quality, body composition, glucose metabolism, and reproductive hormonal profile associated with participation in a 12-week combined exercise program in healthy adult men. **Methods:** Thirty-four healthy men aged 18–24 years were included in a two-arm, non-randomized, parallel-group controlled intervention study and were allocated, according to their willingness and availability rather than by randomization, to either an exercise group (*n* = 17) or a passive control group (*n* = 17). The exercise group completed a supervised combined aerobic and resistance exercise program three times per week for 12 weeks, whereas the control group maintained usual lifestyle habits and did not participate in structured exercise. Outcomes were assessed at baseline and week 12. Body composition, fasting glucose, fasting insulin, HOMA-IR, reproductive hormones, conventional semen parameters, and predefined derived semen indices were analyzed. Conventional semen parameters were the primary outcomes; body composition, glucose-metabolism, and hormonal markers were secondary outcomes; and derived semen indices were exploratory. Time × group interactions were evaluated using two-way mixed repeated-measures ANOVA. Between-group differences in change are reported with 95% confidence intervals (CIs), and a Holm–Bonferroni sensitivity analysis was applied across the 20 interaction tests. The study was registered at ClinicalTrials.gov ([NCT07778576]). **Results:** Significant time × group interactions were observed for BMI, body fat percentage, fasting glucose, fasting insulin, and HOMA-IR, favoring the exercise group. Total testosterone and sex hormone-binding globulin increased, whereas estradiol and prolactin decreased in the exercise group; follicle-stimulating hormone and luteinizing hormone remained unchanged. Semen volume, sperm concentration, total motility, progressive motility, total sperm count, total motile sperm count, and progressive motile sperm count improved in the exercise group compared with the control group (e.g., between-group difference in change in progressive motility: 4.55 percentage points, 95% CI 3.66 to 5.44; total motile sperm count: 5.60 million, 95% CI 2.18 to 9.02). No significant interactions were observed for non-progressive motility or Kruger strict morphology. After Holm–Bonferroni adjustment, the interactions for semen volume, total sperm count, and estradiol were no longer statistically significant. **Conclusions:** A 12-week combined exercise program was associated with favorable metabolic, hormonal, and selected semen-quality changes in healthy adult men. Given the non-randomized design, small sample size, single semen sample at each time point, and absence of fertility endpoints, these findings should be interpreted as preliminary and hypothesis-generating rather than evidence of clinical reproductive benefit.

## 1. Introduction

Male reproductive health is increasingly recognized as a multidimensional outcome shaped not only by genetics and urogenital disease but also by lifestyle, metabolic status, oxidative stress, and endocrine regulation [1,2,3]. Semen analysis remains the principal clinical tool for evaluating male reproductive potential, providing information on sperm concentration, count, motility, and morphology [1,4], although it does not capture sperm DNA integrity, redox balance, or all aspects of fertilization competence [4]. Semen parameters should therefore be regarded as surrogate indicators of male reproductive potential rather than as direct measures of fertility: values within or outside reference ranges do not by themselves determine the probability of conception, which also depends on female-partner factors and on sperm functions that conventional analysis does not assess. Beyond its diagnostic role in infertility, semen quality has also been proposed as a broader marker of general male health, since men with poorer semen parameters show less favorable metabolic, cardiovascular, and mortality-related profiles [5,6].

A growing body of evidence indicates that semen quality may be influenced by modifiable factors related to body composition, metabolic function, oxidative stress, low-grade inflammation, and endocrine regulation. In general male populations, obesity has been associated with lower semen volume, total sperm number, and progressive motility, though not consistently with sperm concentration or morphology [7], and broader syntheses report similarly unfavorable associations between adiposity and semen quality overall [8,9,10]. These associations have been observed alongside disruption of the hypothalamic–pituitary–gonadal axis: cross-sectional imaging studies have found visceral adiposity inversely correlated with bioavailable testosterone [11], and Mendelian randomization analyses suggest genetically predicted higher body mass index is associated with lower sex hormone-binding globulin and bioavailable testosterone and higher estradiol [12]. Insulin resistance, adipokine dysregulation, and oxidative stress have also been proposed as contributing mechanisms [10,13,14]. The endocrine system plays a central regulatory role in this process: follicle-stimulating hormone and luteinizing hormone-stimulated testosterone are described as the principal drivers of spermatogenesis and testicular function, with sex hormone-binding globulin and estradiol further modulating androgen bioavailability and feedback control; prolactin is also implicated, although its specific reproductive role remains less well established [3,15,16].

Regular physical exercise has a plausible biological basis for favorably influencing several of these interconnected pathways. Exercise training is recognized as an effective non-pharmacological strategy for improving insulin sensitivity and glucose regulation, partly through increased GLUT4-mediated glucose uptake and AMPK signaling [17,18], with broader multi-organ metabolic adaptations described beyond skeletal muscle alone [19]. Exercise has also been linked to reductions in systemic inflammatory markers such as CRP and TNF-α, partly through visceral fat loss and myokine-mediated signaling [17,20], and to changes in reproductive hormone responses, although these endocrine adaptations appear to depend on training load, duration, and baseline metabolic status rather than following a single uniform pattern [21]. Because oxidative stress, inflammation, and endocrine imbalance are mechanistically linked to impaired sperm function, these systemic adaptations offer a plausible, though not yet fully established, pathway through which exercise could relate to semen quality.

Observational evidence relating physical activity and sedentary behavior to semen parameters remains mixed. In a large cohort of men screened as potential sperm donors, overall sedentary time was not associated with semen quality, although specific behaviors showed distinct patterns [22]. Time spent cycling has been repeatedly associated with lower sperm concentration or total motile sperm count in fertility-clinic and preconception cohorts [23,24,25], whereas moderate physical activity has been associated with more favorable motility and morphology in healthy young men [26]. A systematic review and meta-analysis similarly concluded that physical activity is associated with semen quality, but that the direction and magnitude of this association vary according to activity type and population [27].

Evidence from exercise-intervention studies is more limited but generally more consistent for moderate, chronic training than for high-intensity or endurance-based regimens. A randomized comparison of three exercise modalities in healthy men found that moderate-intensity continuous training produced more favorable reproductive markers than high-intensity continuous or interval training [28]. In sedentary obese men, structured aerobic training has been associated with improved sperm concentration, motility, morphology, and testosterone concentrations [29], and in obese men with hyperglycemia, a comparable aerobic program improved testosterone, motility, morphology, and sperm DNA fragmentation alongside metabolic markers [30]. A network meta-analysis of randomized trials in fertile and infertile men ranked combined aerobic and resistance training above single-mode programs for measures of male factor infertility, semen quality, and related oxidative and inflammatory markers [31]. In contrast, systematic reviews of endurance athletes report that intense or high-volume training may be associated with less favorable semen parameters, although values often remain within accepted reference ranges [32,33]. Exercise modality may also shape the androgenic response that accompanies these semen findings. Moderate resistance and combined training have generally not been associated with suppressed resting testosterone, whereas chronic high-volume endurance training has been linked to lower resting testosterone in some male athletes, a pattern proposed as one explanation for modality-dependent differences in semen outcomes [21,32,33]. Cycling may additionally involve modality-specific exposures, such as scrotal heat and saddle pressure, that are independent of training load [23,24,25]. Across semen concentration, motility, morphology, and volume, findings therefore diverge depending on training intensity, duration, and population characteristics, indicating that chronic moderate exercise and more intensive training regimens may not act through identical physiological pathways.

Exercise-related changes in reproductive hormones and metabolic markers have often been reported alongside changes in semen parameters in the same intervention studies, suggesting that these outcome domains may be physiologically interconnected. In men with obesity and hyperglycemia, improvements in testosterone and glucose regulation following aerobic training have coincided with improvements in sperm motility, morphology, and DNA fragmentation within the same protocol [30]. In men with metabolic syndrome, combined training has separately been shown to improve waist circumference and insulin-resistance indices [34], consistent with a broader pattern in which exercise-induced metabolic adaptation and reproductive change may share common physiological pathways, although this specific metabolic-syndrome evidence did not itself include semen outcomes.

Several methodological limitations constrain firm conclusions from this literature. Intervention studies specifically addressing male reproductive outcomes remain few and are often based on small samples conducted in heterogeneous populations, including infertile men, obese or hyperglycemic men, and competitive athletes, that may not generalize to healthy, normally active men [35]. Exercise modality, intensity, and duration also vary considerably across studies, complicating comparison across the existing evidence base. A related and more specific limitation, noted in a recent review of the physical-activity–semen-quality literature, is that few controlled exercise trials have assessed body composition, glucose metabolism, hormonal profile, inflammatory status, and a complete panel of conventional and calculated semen indices within a single research framework, with much of the infertility-focused intervention literature drawn from a small number of research groups [35].

Taken together, the available evidence supports a biologically plausible, but incompletely characterized, relationship between structured exercise and male reproductive and metabolic health. What remains less clear is how a combined aerobic and resistance exercise program relates to body composition, glucose metabolism, reproductive hormones, and semen quality when these outcomes are evaluated concurrently, under a controlled design, in healthy men without pre-existing obesity, hyperglycemia, or infertility. Healthy, normal-weight young men were selected deliberately for three reasons. First, most previous intervention studies enrolled obese, hyperglycemic, infertile, or athletic men [29,30,31,32,33,35], whose findings cannot be assumed to apply to men without metabolic or reproductive impairment, in whom smaller changes might be expected. Second, a metabolically healthy and age-homogeneous sample reduces confounding by disease-related endocrine disturbance, age-related decline in testosterone and semen quality, and medication use. Third, this population allows examination of exercise as a preventive lifestyle factor in male reproductive physiology rather than as a therapy for an existing disorder. The present study was therefore designed to examine changes associated with a 12-week combined exercise intervention, relative to a passive control condition, in body composition, glucose metabolism, hormonal profile, and conventional and calculated semen quality indices in healthy men. It was hypothesized that the exercise intervention would be associated with more favorable changes over time in these interconnected outcome domains compared with the control condition, consistent with the physiological pathways described above.

## 2. Materials and Methods

### 2.1. Study Design

This study was designed as a 12-week, two-arm, parallel-group non-randomized controlled intervention study to examine changes in body composition, glucose metabolism, hormonal profile, and semen quality associated with a combined exercise program in healthy adult men. Participants were assigned to the exercise or control group based on their willingness and availability to participate in the supervised exercise program. No random-allocation procedure was used. Because allocation depended on participants’ self-selection, individuals in the exercise group may have differed systematically from controls in motivation, habitual physical activity, health behaviors, sleep, diet, or other unmeasured characteristics. This potential selection bias cannot be removed by statistical adjustment and is considered central to the interpretation of all findings. The exercise group completed a supervised combined exercise intervention, whereas the control group maintained their usual lifestyle habits and did not participate in any structured exercise program during the study period.

**Figure 1:**
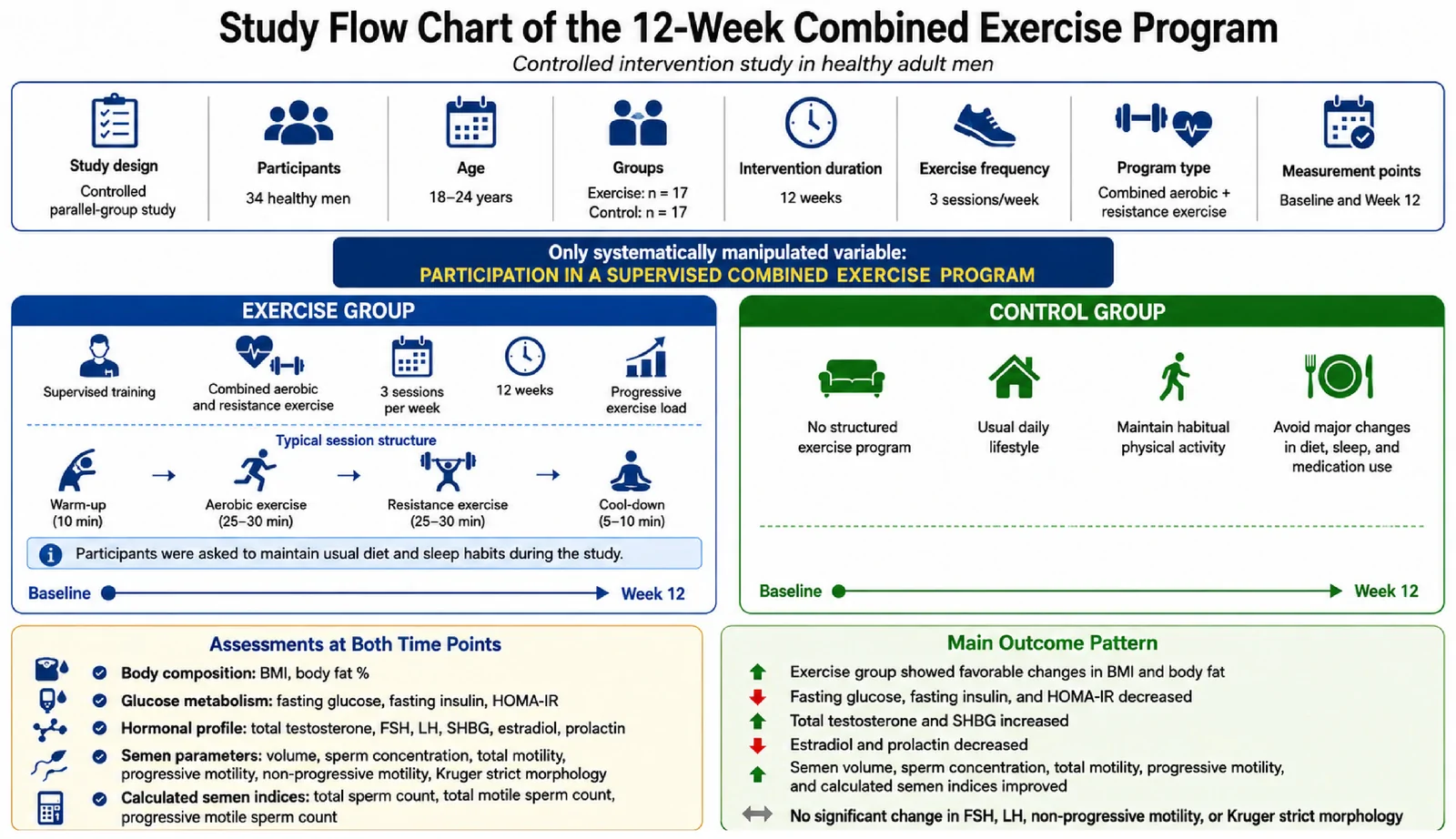
Study Flow and Overview of the 12-Week Combined Exercise Intervention.

Measurements were performed at two time points: baseline, before the intervention, and week 12, after completion of the intervention period. Outcomes were classified a priori into three levels. Primary outcomes were the conventional semen parameters (semen volume, sperm concentration, total motility, progressive motility, non-progressive motility, and Kruger strict morphology). Secondary outcomes were body composition (BMI and body fat percentage), glucose-metabolism markers (fasting glucose, fasting insulin, and HOMA-IR), and the reproductive hormonal profile (total testosterone, follicle-stimulating hormone, luteinizing hormone, sex hormone-binding globulin, estradiol, and prolactin). The calculated semen quality indices (total sperm count, total motile sperm count, and progressive motile sperm count) were treated as exploratory outcomes because they are arithmetically derived from the primary semen parameters and therefore represent integrated summary measures rather than independent endpoints. Body mass and fat-free mass, which were added during peer review to characterize the nature of the body-composition changes, were treated as additional exploratory outcomes outside the prespecified hierarchy and were not included in the 20-outcome multiplicity family described in Section 2.7. The primary analytical focus was the time × group interaction, which was used to determine whether changes from baseline to week 12 differed between the exercise and control groups (Figure 1). The study was registered at ClinicalTrials.gov (https://clinicaltrials.gov/study/NCT07778576) “URL (last accessed on 26 October 2026)”.

### 2.2. Participants

A total of 34 healthy adult men participated in the study. Seventeen participants were included in the exercise group and 17 participants were included in the control group. Participants were between 18 and 24 years of age. Age, height, and baseline body mass were 20.12 ± 1.90 years, 178.18 ± 4.69 cm, and 70.44 ± 4.94 kg, respectively, in the exercise group (*n* = 17), and 19.59 ± 1.58 years, 178.53 ± 3.78 cm, and 71.57 ± 4.77 kg, respectively, in the control group (*n* = 17). Between-group comparisons using Welch’s independent-samples *t*-tests revealed no statistically significant differences in age (*p* = 0.384), height (*p* = 0.811), or baseline body mass (*p* = 0.499).

Participants were recruited from healthy young adult men who volunteered to participate in the study. The inclusion criteria were male sex, age between 18 and 24 years, apparently good general health, absence of known reproductive, metabolic, endocrine, cardiovascular, or chronic systemic disease, ability to participate safely in a structured exercise program, and provision of written informed consent.

The exclusion criteria included a history of infertility treatment, previous varicocele surgery, urogenital infection, endocrine disorders, diabetes mellitus or other metabolic diseases, use of medications or supplements that could affect reproductive or metabolic outcomes, use of anabolic steroids or hormonal therapy, and musculoskeletal conditions that could limit participation in the exercise intervention.

Participants were instructed to maintain their usual lifestyle habits throughout the 12-week study period and to avoid major changes in diet, sleep habits, medication use, and habitual physical activity. The exercise group participated in the supervised exercise intervention, whereas the control group was instructed not to undertake a structured exercise program during the study period.

At baseline, participants were characterized according to age, height, and body mass. Habitual physical activity was not assessed using a standardized instrument, and smoking status, alcohol consumption, and usual sleep duration were not systematically recorded. Consequently, these lifestyle characteristics could not be quantitatively compared between the exercise and control groups.

Participants were also required to complete the baseline assessment, including body composition assessment, blood sampling, and semen analysis.

Participants were also excluded if they failed to comply with the semen collection procedure, including the required 3-day sexual abstinence period.

**Figure 2:**
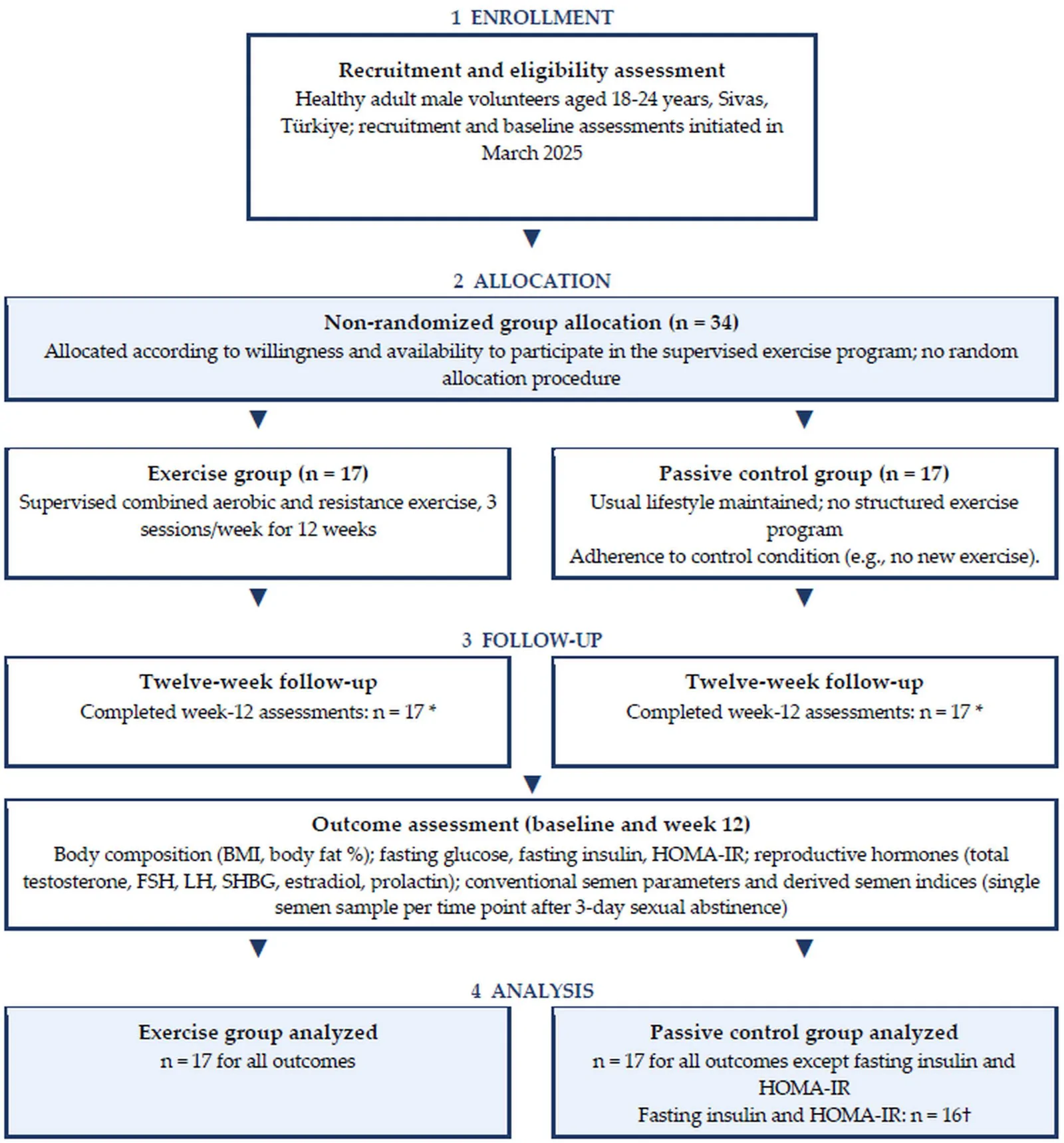
TREND participant flow diagram. † One control-group observation was missing for fasting insulin and HOMA-IR; these outcomes were analyzed using complete-case analysis. * All 34 participants completed the intervention and the post-intervention assessments and were included in the final analyses.

Before participation, all individuals were informed about the study procedures, potential risks, and requirements, and written informed consent was obtained. Participants were assigned to groups according to their willingness and availability to participate in the supervised exercise program. No random-allocation procedure was used. As shown in Figure 2, the study flow was conducted in accordance with the TREND guidelines, and the completed TREND checklist is provided in the Supplementary Materials.

A total of 34 eligible men were enrolled and allocated non-randomly to the exercise group (*n* = 17) or the control group (*n* = 17). No participant was lost to follow-up during the 12-week study period. However, one control-group value was missing for fasting insulin and HOMA-IR, and these analyses therefore included 17 participants in the exercise group and 16 in the control group. Given the non-randomized allocation, the baseline comparability of the groups was assessed for all outcome variables (Section 3).

### 2.3. Combined Exercise Intervention

Participants in the exercise group completed a supervised 12-week combined exercise program consisting of aerobic and resistance exercise components. The intervention was performed three days per week on non-consecutive days, preferably Monday, Wednesday, and Friday, to allow adequate recovery between sessions. Each training session lasted approximately 60–70 min and included a warm-up phase, aerobic exercise, resistance exercise, and a cool-down phase. The program was designed according to the principles of progressive overload, individual tolerance, and gradual adaptation. The 12-week duration was selected because it exceeds the approximately 74 days classically estimated for one complete cycle of human spermatogenesis and epididymal transit [36], so that spermatozoa ejaculated at the post-intervention assessment would have developed largely during the intervention period, and because 12 weeks is a commonly used duration for combined-training interventions in which changes in body composition and insulin sensitivity can be detected [37,38,39]. The program was designed as a health-oriented conditioning intervention combining cardiorespiratory and muscular strength-endurance stimuli rather than as a hypertrophy- or performance-focused protocol; moderate aerobic intensities and moderate resistance loads (50–75% of one-repetition maximum, 8–15 repetitions) were chosen to reflect general exercise recommendations for healthy adults and to avoid the high-volume or high-intensity training loads that have been associated with less favorable semen parameters [32,33].

Each session began with a 10 min warm-up consisting of low-intensity aerobic activity, dynamic mobility exercises, and joint range-of-motion movements. The warm-up aimed to gradually increase heart rate, prepare the major muscle groups for exercise, and reduce the risk of musculoskeletal discomfort during the main training phase.

The aerobic component was performed for 25–30 min per session using treadmill walking/running. During the first four weeks, aerobic exercise was performed at moderate intensity, corresponding approximately to 50–60% of heart rate reserve or 60–65% of age-predicted maximal heart rate. During weeks 5–8, the intensity was increased to approximately 60–70% of heart rate reserve or 65–75% of age-predicted maximal heart rate. During weeks 9–12, participants exercised at approximately 65–75% of heart rate reserve or 70–80% of age-predicted maximal heart rate. Participants were also instructed to exercise within a target rating of perceived exertion of 11–14 during the main aerobic phase. Participants were encouraged to maintain the prescribed intensity without reaching exhaustion. All participants in the exercise group performed aerobic exercise exclusively on a treadmill throughout the 12-week intervention. No alternative aerobic exercise modalities, such as stationary cycling or elliptical exercise, were used during the supervised sessions.

The resistance exercise component lasted approximately 25–30 min and included exercises targeting the major muscle groups of the lower body, upper body, and trunk. The main resistance exercises included leg press or squat, knee extension, hamstring curl, chest press or push-up, seated row, lat pull-down, shoulder press, abdominal crunch, and plank exercises. During weeks 1–4, participants performed 1–2 sets of 12–15 repetitions at low-to-moderate intensity to ensure proper technique and neuromuscular adaptation. During weeks 5–8, the volume was increased to 2–3 sets of 10–12 repetitions. During weeks 9–12, participants performed 3 sets of 8–12 repetitions with a moderate training load. Resistance intensity was adjusted to approximately 50–60% of one-repetition maximum during the initial phase, 60–70% during the middle phase, and 65–75% during the final phase. When direct one-repetition maximum testing was not feasible, load progression was based on participant tolerance, correct exercise technique, and perceived exertion. Rest intervals between sets and exercises were kept between 60 and 90 s.

Training progression was applied gradually throughout the 12-week period. Increases in exercise intensity or volume were made only when participants could complete the prescribed workload with proper technique and without excessive fatigue or discomfort. The first four weeks were considered the adaptation phase, the second four weeks the progression phase, and the final four weeks the maintenance/intensification phase. Exercise sessions were supervised by trained personnel to ensure correct technique, adherence to the protocol, and participant safety.

At the end of each session, participants completed a 5–10 min cool-down consisting of low-intensity walking and static stretching exercises for the major muscle groups. Participants were instructed to report any pain, discomfort, illness, or absence during the intervention period. They were encouraged to attend all scheduled training sessions. The intervention was scheduled to include 36 supervised exercise sessions over 12 weeks, with three sessions planned per week. However, individual session attendance was not systematically recorded; therefore, the attendance rate, mean number of sessions attended, and range of attendance could not be calculated. Similarly, exercise intensity was not systematically monitored or documented using heart-rate monitoring or ratings of perceived exertion (RPE). Consequently, individual adherence to the prescribed exercise intensity could not be quantified.

The control group did not receive any structured exercise intervention during the 12-week study period. Participants in this group were instructed to maintain their usual daily lifestyle habits and avoid initiating a new exercise program. Participants in both groups were asked to avoid major changes in diet, sleep habits, medication use, supplement intake, and habitual physical activity throughout the study period. However, these lifestyle factors were not systematically quantified or objectively monitored during the intervention. Consequently, adherence to these instructions could not be independently verified, and potential changes in lifestyle factors are acknowledged as possible sources of confounding (Section 4.2).

### 2.4. Body Composition Measurements

Body composition measurements were performed at baseline and week 12 using a Tanita bioelectrical impedance analysis (BIA) device. All measurements were conducted in the morning after an overnight fast and bladder voiding. Participants were instructed to refrain from strenuous physical exercise for at least 24 h before each assessment. Hydration status was assessed as part of the measurement procedure. These standardized pre-measurement conditions were applied to minimize potential variation in BIA-derived body composition estimates.

Height was measured using a stadiometer, with participants standing barefoot in an upright position. Body mass was measured with participants wearing light clothing and no shoes. Body mass index (BMI) was calculated as body mass in kilograms divided by height in meters squared (kg/m^2^). Body fat percentage was assessed using the Tanita BIA (Tanita BC-418 MA, Tanita Corporation, Tokyo, Japan) device at both measurement time points.

Fat-free mass was obtained from the same BIA device. The body composition outcomes included BMI, body fat percentage, body mass, and fat-free mass.

### 2.5. Blood Sampling and Biochemical Analyses

Venous blood samples were collected at baseline and at week 12 in the morning following a 12 h overnight fast. Blood samples were obtained from the antecubital vein with participants in a seated position. Approximately 5 mL of venous blood was collected into serum separator (gel) tubes. Samples were centrifuged at 3000 rpm for 10 min to separate serum. These procedures were consistent with previously reported blood collection and sample processing procedures from the same laboratory.

All biochemical and hormonal analyses were performed at the Biochemistry Laboratory of Sivas Cumhuriyet University Health Services Application and Research Hospital, Sivas, Türkiye. Fasting glucose concentrations were measured using a photometric enzymatic method on an automated clinical chemistry analyzer (Roche Cobas 8000 c702, Roche Diagnostics GmbH, Mannheim, Germany). Fasting insulin and hormonal parameters were measured using an automated electrochemiluminescence immunoassay analyzer (Roche Cobas 8000 e801, Roche Diagnostics GmbH, Mannheim, Germany), in accordance with the manufacturers’ instructions and routine laboratory procedures.

Insulin resistance was estimated using the homeostatic model assessment of insulin resistance (HOMA-IR), calculated as follows:

HOMA-IR = [fasting glucose (mg/dL) × fasting insulin (µIU/mL)]/405.

The hormonal profile included total testosterone, follicle-stimulating hormone (FSH), luteinizing hormone (LH), sex hormone-binding globulin (SHBG), estradiol, and prolactin. These parameters were assessed to characterize the hormonal profile in relation to male reproductive physiology and the hypothalamic–pituitary–gonadal axis.

### 2.6. Semen Collection and Analysis

Semen samples were collected at baseline and week 12 under standardized conditions. Participants were instructed to maintain a 3-day period of sexual abstinence before each semen collection. Samples were obtained by masturbation into sterile, wide-mouth, non-toxic containers and were delivered immediately to the laboratory for analysis. Participants were instructed to report any sample loss during collection.

After complete liquefaction at room temperature, semen samples were analyzed by trained laboratory personnel according to standardized semen analysis procedures and the recommendations of the WHO Laboratory Manual for the Examination and Processing of Human Semen (6th edition, 2021) [40]. Semen volume was measured in milliliters using the graduated collection container.

Sperm concentration and motility parameters were assessed using a Makler Counting Chamber (Sefi Medical Instruments Ltd., Haifa, Israel) under a light microscope. An aliquot of well-mixed semen was placed on the chamber according to the manufacturer’s instructions, and sperm concentration was expressed as million/mL. Sperm motility was evaluated microscopically and classified as total motility, progressive motility, and non-progressive motility. Total motility was calculated as the sum of progressive and non-progressive motility.

Sperm morphology was evaluated according to Kruger strict morphology criteria following sperm smear preparation and staining [41]. Morphologically normal spermatozoa were expressed as a percentage. Conventional semen parameters included semen volume, sperm concentration, total motility, progressive motility, non-progressive motility, and Kruger strict morphology.

In addition to conventional semen parameters, calculated semen quality indices were derived. Total sperm count was calculated by multiplying semen volume by sperm concentration:

Total sperm count (million) = semen volume (mL) × sperm concentration (million/mL).

Total motile sperm count was calculated by multiplying total sperm count by total motility percentage and dividing by 100:

Total motile sperm count (million) = total sperm count × total motility (%)/100.

Progressive motile sperm count was calculated by multiplying total sperm count by progressive motility percentage and dividing by 100:

Progressive motile sperm count (million) = total sperm count × progressive motility (%)/100.

These calculated indices were used to provide integrated information on sperm quantity and motility; however, they were derived variables rather than independently measured semen parameters. Only one semen sample was obtained at each time point. Given the substantial within-subject variability of semen parameters, this limitation was considered when interpreting the results (Section 4.2).

### 2.7. Statistical Analysis

All statistical analyses, including the supplementary sensitivity analyses, were performed using IBM SPSS Statistics for Windows, version 32.0 (IBM Corp., Armonk, NY, USA). Data were summarized as mean ± standard deviation (SD). Baseline demographic characteristics and outcome values were compared between groups using independent-samples *t*-tests. Statistical assumptions were evaluated before inferential analyses. Normality was assessed using the Shapiro–Wilk test applied to change scores separately by group and to the ANCOVA residuals. Homogeneity of variance was evaluated using the median-centered Levene’s test applied to change scores and, for the ANCOVA models, the Breusch–Pagan test. Potentially influential observations were screened using externally studentized residuals (screening threshold |r| > 3) and Cook’s distance (screening threshold 4/*N*). These diagnostic measures were used to identify observations requiring scrutiny rather than as automatic criteria for exclusion.

A two-way mixed repeated-measures analysis of variance (ANOVA) was used to evaluate the effects of time, group, and the time × group interaction. Time was specified as the within-subject factor, with two levels (baseline and week 12), and group was specified as the between-subject factor, with two levels (exercise and control). The principal analytical focus was the time × group interaction, which tested whether the change from baseline to week 12 differed between groups. Partial eta squared (η*p*^2^) was reported as the effect size for ANOVA results. Because the within-subject factor comprised only two time points, the sphericity assumption was not applicable. The between-group difference in mean change (exercise minus control), together with its 95% confidence interval (CI), was calculated for each outcome.

Because the diagnostic assessment indicated that the assumptions were not uniformly satisfied across all outcomes, additional sensitivity analyses were conducted using Welch’s independent-samples *t*-test and the Mann–Whitney U test to compare individual change scores between groups. Change scores were calculated as week 12 minus baseline values. These analyses were used to assess the robustness of the findings to potential heterogeneity of variance and departures from normality. No participant was excluded solely on the basis of an influence-diagnostic threshold.

To account for the non-randomized group allocation, analysis of covariance (ANCOVA) was additionally examined as a sensitivity analysis, with the week 12 value as the dependent variable, group as the fixed factor, and the corresponding baseline value as the covariate. The homogeneity-of-regression-slopes assumption was assessed by examining the group × baseline interaction. ANCOVA-adjusted group estimates were interpreted cautiously when this assumption was not satisfied and were not considered definitive evidence of an intervention effect for those outcomes. Because residual heteroscedasticity was detected for several outcomes, ANCOVA confidence intervals and *p*-values were estimated using HC3 heteroscedasticity-robust standard errors, and conventional estimates are reported alongside them. For these supplementary analyses, Benjamini–Hochberg q-values were calculated separately within each test family (Welch, Mann–Whitney, and HC3 ANCOVA) across all 22 reported outcomes, including body mass and fat-free mass. The results of the Welch, Mann–Whitney, and ANCOVA sensitivity analyses, together with the corresponding assumption and influence diagnostics, are reported in Supplementary Tables S1–S4.

Sperm concentration was analyzed using log10(x + 1)-transformed values because of its positively skewed distribution, whereas descriptive statistics were presented in the original units. The remaining outcomes were analyzed using their original values in the primary analyses, with sensitivity analyses used to assess the influence of departures from model assumptions. Calculated semen quality indices were treated as derived outcomes based on semen volume, sperm concentration, and motility percentages.

Because multiple outcomes were examined, the original time × group interaction *p*-values were treated as nominal. As sensitivity analyses for multiplicity, the Holm–Bonferroni procedure, which controls the family-wise error rate, and the Benjamini–Hochberg procedure, which controls the false discovery rate, were applied across the 20 time × group interaction tests for the prespecified outcomes. The adjusted *p*-values were calculated from the corresponding unadjusted interaction *p*-values. Secondary and exploratory outcomes were interpreted as hypothesis-generating, and statistical findings were considered alongside effect sizes, confidence intervals, and the limitations of the study design.

Due to one missing control-group value, fasting insulin and HOMA-IR analyses were conducted using complete-case analysis, with 17 participants in the exercise group and 16 participants in the control group. All other analyses included 17 participants in each group. Statistical significance was set at *p* < 0.05 using two-sided tests.

An a priori sample-size calculation was performed using G*Power software, version 3.1.9.7 (Heinrich Heine University Düsseldorf, Düsseldorf, Germany). The calculation was based on an F test for the within–between interaction in a repeated-measures ANOVA with two groups and two measurements. Assuming a medium effect size (f = 0.25), an alpha level of 0.05, statistical power of 0.80, a correlation of 0.50 between repeated measurements, and a nonsphericity correction of 1, the estimated minimum total sample size was 34 participants. The assumed effect size was based on Cohen’s conventional medium-effect benchmark rather than an outcome-specific pilot estimate. Accordingly, the sample-size calculation should not be interpreted as demonstrating adequate statistical power for each secondary or exploratory outcome.

## 3. Results

All 34 participants completed the 12-week intervention and post-intervention assessments. No exercise-related adverse events were reported during the intervention period. Baseline comparisons using independent-samples *t*-tests revealed no statistically significant between-group differences in the assessed outcome variables (all *p* ≥ 0.100). Nevertheless, numerical differences were observed in some semen parameters, particularly semen volume (2.10 ± 1.22 vs. 1.58 ± 1.36 mL; *p* = 0.247) and non-progressive motility (5.59 ± 1.66% vs. 8.12 ± 5.94%; *p* = 0.100) in the exercise and control groups, respectively. Neither difference reached statistical significance.

For body composition parameters, significant time × group interactions were observed for BMI and body fat percentage. BMI decreased in the exercise group from 22.19 ± 1.41 to 21.62 ± 1.34 kg/m^2^, while it remained nearly unchanged in the control group from 22.45 ± 1.27 to 22.46 ± 1.23 kg/m^2^, F(1, 32) = 282.726, *p* < 0.001, η*p*^2^ = 0.898. Similarly, body fat percentage decreased in the exercise group from 15.10 ± 3.05% to 13.78 ± 2.71%, whereas the control group remained stable from 15.56 ± 2.71% to 15.64 ± 2.67%, F(1, 32) = 167.586, *p* < 0.001, η*p*^2^ = 0.840 (Table 1).

Body mass decreased in the exercise group from 70.44 ± 4.94 to 68.62 ± 4.70 kg, whereas it remained stable in the control group, changing from 71.57 ± 4.77 to 71.62 ± 4.78 kg. The time × group interaction was significant, F(1, 32) = 327.334, *p* < 0.001, η*p*^2^ = 0.911. In contrast, fat-free mass changed from 59.42 ± 4.15 to 59.12 ± 3.64 kg in the exercise group and from 60.33 ± 2.80 to 60.35 ± 2.83 kg in the control group, with no significant time × group interaction, F(1, 32) = 0.849, *p* = 0.364, η*p*^2^ = 0.026. However, this mean-based result was strongly influenced by a single exercise-group observation, and the rank-based sensitivity analysis indicated lower fat-free-mass change scores in the exercise group than in the control group (Mann–Whitney *p* < 0.001; Supplementary Tables S1 and S3); the fat-free-mass result should therefore be interpreted with caution.

The between-group differences in mean change (exercise minus control) were −0.58 kg/m^2^ (95% CI: −0.65 to −0.51) for BMI, −1.40 percentage points (95% CI: −1.62 to −1.18) for body fat percentage, −1.86 kg (95% CI: −2.06 to −1.65) for body mass, and −0.32 kg (95% CI: −1.03 to 0.39) for fat-free mass (Table 1).

Significant time × group interactions were observed for fasting glucose, fasting insulin, and HOMA-IR. In the exercise group, fasting glucose decreased from 90.71 ± 5.44 to 87.44 ± 5.13 mg/dL, fasting insulin decreased from 8.18 ± 4.00 to 6.80 ± 3.25 µIU/mL, and HOMA-IR decreased from 1.85 ± 0.95 to 1.48 ± 0.74. In contrast, these parameters remained largely unchanged in the control group. The interaction effects were significant for fasting glucose, F(1, 32) = 107.397, *p* < 0.001, η*p*^2^ = 0.770; fasting insulin, F(1, 31) = 39.631, *p* < 0.001, η*p*^2^ = 0.561; and HOMA-IR, F(1, 31) = 43.205, *p* < 0.001, η*p*^2^ = 0.582 (Table 2). Between-group differences in mean change were −3.48 mg/dL (95% CI −4.16 to −2.80) for fasting glucose, −1.32 µIU/mL (95% CI −1.75 to −0.89) for fasting insulin, and −0.36 (95% CI −0.47 to −0.25) for HOMA-IR.

For hormonal profile, significant time × group interactions were observed for total testosterone, SHBG, estradiol, and prolactin, whereas no significant interaction was found for FSH or LH. In the exercise group, total testosterone increased from 624.46 ± 72.51 to 681.93 ± 83.40 ng/dL and SHBG increased from 34.33 ± 3.08 to 35.98 ± 3.22 nmol/L. Estradiol decreased from 25.03 ± 2.33 to 24.08 ± 2.49 pg/mL, and prolactin decreased from 9.59 ± 1.40 to 9.08 ± 1.43 ng/mL. In contrast, these hormones remained largely unchanged in the control group. The interaction effects were significant for total testosterone, F(1, 32) = 203.808, *p* < 0.001, η*p*^2^ = 0.864; SHBG, F(1, 32) = 19.131, *p* < 0.001, η*p*^2^ = 0.374; estradiol, F(1, 32) = 5.508, *p* = 0.025, η*p*^2^ = 0.147; and prolactin, F(1, 32) = 17.887, *p* < 0.001, η*p*^2^ = 0.359. No significant time × group interactions were detected for FSH, F(1, 32) = 0.808, *p* = 0.376, η*p*^2^ = 0.025, or LH, F(1, 32) = 0.056, *p* = 0.815, η*p*^2^ = 0.002 (Table 3). Between-group differences in mean change were 57.75 ng/dL (95% CI 49.51 to 65.99) for total testosterone, 1.66 nmol/L (95% CI 0.89 to 2.43) for SHBG, −0.69 pg/mL (95% CI −1.29 to −0.09) for estradiol, and −0.59 ng/mL (95% CI −0.87 to −0.31) for prolactin.

Significant time × group interactions were observed for semen volume, sperm concentration, total motility, progressive motility, and all calculated semen quality indices. In the exercise group, semen volume increased from 2.10 ± 1.22 to 2.16 ± 1.22 mL, sperm concentration increased from 22.88 ± 15.72 to 25.57 ± 17.18 million/mL, total motility increased from 40.53 ± 12.24% to 44.52 ± 11.74%, and progressive motility increased from 34.94 ± 12.78% to 38.46 ± 12.13%. Similarly, total sperm count increased from 50.56 ± 59.16 to 58.38 ± 66.59 million, total motile sperm count increased from 19.68 ± 23.65 to 25.56 ± 30.36 million, and progressive motile sperm count increased from 17.02 ± 20.80 to 21.79 ± 25.36 million. In contrast, the control group showed only minimal changes or slight decreases in these parameters. The interaction effects were significant for semen volume, F(1, 32) = 5.241, *p* = 0.029, η*p*^2^ = 0.141; sperm concentration, F(1, 32) = 46.928, *p* < 0.001, η*p*^2^ = 0.595; total motility, F(1, 32) = 112.707, *p* < 0.001, η*p*^2^ = 0.779; progressive motility, F(1, 32) = 107.672, *p* < 0.001, η*p*^2^ = 0.771; total sperm count, F(1, 32) = 7.106, *p* = 0.012, η*p*^2^ = 0.182; total motile sperm count, F(1, 32) = 11.112, *p* = 0.002, η*p*^2^ = 0.258; and progressive motile sperm count, F(1, 32) = 16.413, *p* < 0.001, η*p*^2^ = 0.339. No significant time × group interactions were found for non-progressive motility, F(1, 32) = 0.762, *p* = 0.389, η*p*^2^ = 0.023, or Kruger strict morphology, F(1, 32) = 1.000, *p* = 0.325, η*p*^2^ = 0.030 (Table 4). Between-group differences in mean change were 0.06 mL (95% CI 0.01 to 0.11) for semen volume, 4.85 percentage points (95% CI 3.92 to 5.78) for total motility, 4.55 percentage points (95% CI 3.66 to 5.44) for progressive motility, 6.19 million (95% CI 1.46 to 10.92) for total sperm count, 5.60 million (95% CI 2.18 to 9.02) for total motile sperm count, and 4.64 million (95% CI 2.31 to 6.97) for progressive motile sperm count; because sperm concentration was analyzed on the log scale, a confidence interval in original units is not reported for this variable. Because total sperm count, total motile sperm count, and progressive motile sperm count are arithmetic products of semen volume, sperm concentration, and motility, their interactions are not independent of those of their component variables and should not be regarded as additional, independent evidence.

In the multiplicity sensitivity analysis (Holm–Bonferroni procedure across the 20 interaction tests), the interactions for semen volume (adjusted *p* = 0.152), estradiol (adjusted *p* = 0.152), and total sperm count (adjusted *p* = 0.084) were no longer statistically significant, whereas all interactions with nominal *p* ≤ 0.002 remained significant (adjusted *p* ≤ 0.018). Under false-discovery-rate control (Benjamini–Hochberg procedure), all nominally significant interactions remained below 0.05 (largest adjusted *p* = 0.036). Interactions with nominal *p*-values close to 0.05 should therefore be interpreted with particular caution.

The sensitivity analyses of change scores (Supplementary Table S1) reproduced the between-group differences in the primary analyses. All interactions that were nominally significant in the primary analysis remained significant in Welch’s *t*-tests (all *p* ≤ 0.034) and in the Mann–Whitney U tests, with the exception of semen volume, for which the rank-based comparison was not significant (*p* = 0.138; q = 0.169). For fat-free mass, Welch’s *t*-test was not significant (*p* = 0.370), whereas the Mann–Whitney U test was (*p* < 0.001). In the baseline-adjusted ANCOVA (Supplementary Table S2), adjusted group differences were similar in direction and magnitude to the unadjusted estimates, and all nominally significant interactions remained significant with HC3 robust inference (all *p* ≤ 0.035). However, the homogeneity-of-regression-slopes assumption was not supported (group × baseline *p* < 0.05) for body fat percentage, body mass, fasting insulin, HOMA-IR, sperm concentration, Kruger strict morphology, total sperm count, total motile sperm count, and progressive motile sperm count; for these outcomes, the ANCOVA estimates are presented as screening estimates only. Diagnostic checks (Supplementary Tables S3 and S4) indicated departures from normality or homoscedasticity for several outcomes, particularly the derived sperm-count indices, and identified influential observations; no observation was excluded on the basis of these diagnostics.

## 4. Discussion

This 12-week controlled combined exercise intervention was associated with favorable changes in body composition, glucose metabolism, most components of the reproductive hormonal profile, and several conventional and calculated semen indices in healthy men. Follicle-stimulating hormone, luteinizing hormone, non-progressive motility, and sperm morphology did not change. These results should be read as an exploratory, hypothesis-generating pattern rather than confirmed clinical effects, given the study design and sample size discussed below.

The increase in total and progressive motility is consistent with the randomized controlled trial most comparable to the present study in population, which found that moderate-intensity continuous training improved reproductive markers in healthy men over 24 weeks [28]. Directionally similar motility gains have been reported in aerobic-training trials in obese and hyperglycemic men [29,30], although these populations differ metabolically from the present sample. The concentration increase observed here is directionally consistent with Rosety et al. [29], but Ismail et al. [30], despite reporting improved motility, morphology, testosterone, and DNA fragmentation, found no significant change in semen volume or sperm count over 14 weeks and attributed this absence specifically to insufficient duration. That study should not be read as supporting a concentration or volume increase, since it did not report one. Against this backdrop, the present study’s detection of significant concentration and volume changes at only 12 weeks is a genuine point of divergence from comparable literature and warrants replication before being treated as a robust or fast-acting effect.

The semen-volume finding deserves particular caution. Several observational studies and a meta-analysis of randomized controlled trials have found no meaningful association between exercise or physical activity and semen volume specifically [24,42,43,44], and even Maleki et al. [28] required the full 24-week protocol before volume changed. The volume increase reported here was numerically small, and its interaction *p*-value was among the closest to the conventional significance threshold in this dataset. It should therefore be interpreted as requiring independent confirmation rather than as an established exercise effect. Moreover, this interaction did not remain significant after Holm–Bonferroni adjustment, and its 95% confidence interval (0.01 to 0.11 mL) extended to changes too small to be clinically meaningful. The semen-volume difference was also not supported by the rank-based Mann–Whitney sensitivity analysis (*p* = 0.138; Supplementary Table S1).

Morphology and non-progressive motility showed no significant time × group interaction. These null results are substantive rather than incidental. Prior evidence on exercise and morphology is itself divided: some trials and pooled analyses report improvement with aerobic or resistance training [29,44], including Ismail et al. [30], who found a morphology benefit despite no volume or count change, essentially the reverse pattern to the one observed here. Other studies report null or unfavorable morphology responses under comparable moderate-exercise conditions [45], and an observational cohort has described a non-linear relationship in which only a specific activity range is associated with better morphology, with both low and vigorous activity performing less favorably [26]. Several explanations may account for the absence of a morphology effect here. Strict-criteria morphology scoring shows limited variability and known observer-dependent measurement error, which can obscure small true changes. Morphological differentiation occurs across a developmental window: human spermatogenesis and epididymal transit require approximately 74 days by classical estimation [36], so a 12-week intervention approximates, but does not clearly exceed, one complete cycle. A single post-intervention semen sample cannot capture whether a delayed morphological change was beginning to emerge. None of these explanations can be confirmed from the present data, and the baseline morphology values themselves should not be classified as clinically abnormal without reference to individual-level data and the applicable WHO decision limits, which were not reported here [40]. Non-progressive motility is rarely analyzed as an independent outcome in this literature, with most studies reporting only total or progressive motility; its null result is therefore not clearly anomalous.

The calculated indices, namely total sperm count, total motile sperm count, and progressive motile sperm count, followed the same direction as their constituent conventional parameters. This is expected, since these indices are arithmetic products of volume, concentration, and motility rather than independently measured biological quantities. They should be understood as integrated summaries of the conventional parameters already discussed, which does not diminish their established relevance in andrological practice, but does mean that their significance here does not constitute separate corroborating evidence beyond what has already been described.

Body composition and glucose-metabolism changes were directionally consistent with a substantial combined-training literature, including pooled reductions in HOMA-IR reported for men specifically [17], superior insulin-sensitivity gains from combined versus single-mode training in a well-known randomized controlled trial [46], and comparable 12-week combined-training trials reporting parallel improvements in adiposity and insulin resistance [37,38,39]. Nearly all of this comparator literature, however, was generated in overweight, obese, or metabolically impaired men, whereas the present sample’s baseline values were already within commonly accepted normal ranges. Achieving further large reductions from an already normal metabolic baseline is less well precedented, and the very large effect sizes observed for several of these variables warrant cautious interpretation given the small sample, the non-randomized study design, and the absence of a formal correction for multiple testing.

The hormonal results require similarly careful reading. The testosterone increase is consistent with several reviews and trials reporting exercise-associated testosterone elevation in varied male populations [29,47,48,49], and the concurrent SHBG increase aligns with reports that SHBG rises with training largely independent of modality [47]. A well-powered 12-month randomized controlled trial in a comparable sedentary population, however, found that exercise increased SHBG but produced no significant change in total or free testosterone or estradiol [50], and a meta-analysis of exercise added to hypocaloric diets found no incremental hormonal benefit from exercise beyond diet alone [51]. These findings sit in tension with the magnitude and consistency of the present testosterone, SHBG, and estradiol changes over a considerably shorter period, and this discrepancy should be weighed against the present study’s small sample rather than read as evidence of an unusually strong or fast hormonal response. An important limitation applies to all of these hormonal interpretations: free testosterone, calculated free testosterone, albumin, and the free androgen index were not assessed. Without an estimate of biologically available androgen, the physiological meaning of a concurrent rise in total testosterone and SHBG cannot be fully determined, since SHBG binds testosterone and can leave the bioavailable fraction essentially unchanged even when total testosterone rises. The estradiol decrease was small, did not remain significant after adjustment for multiple testing, and should not be attributed to a specific mechanism, such as reduced peripheral aromatization, because neither aromatase activity nor mediation was assessed. The prolactin decrease should be treated as exploratory and of uncertain clinical relevance; basal prolactin has been reported as similar between trained and untrained men in several studies even when stimulated responses differ [52,53], and one long-duration exercise intervention found no change in serum prolactin at either 3 or 12 months, albeit in a female population [54]. Prolactin secretion is also sensitive to circadian timing, psychological stress, recent physical exertion, and its own pulsatile release pattern, any of which could have influenced a single measurement at each time point; therefore, a small reduction in prolactin should not be read as evidence of improved reproductive function.

The stability of FSH and LH is compatible with a substantial body of evidence indicating that gonadotropin secretion is comparatively resistant to short-term exercise exposure, with FSH generally described as unresponsive to training and LH showing, at most, modest change under extreme or prolonged endurance exposure [55,56,57,58]. Some conflicting evidence exists: a comparable 12-week concurrent-training trial in obese men reported a significant FSH increase alongside a significant LH decrease [59], and a cross-sectional comparison found higher FSH and LH in physically active men [60]. On balance, the present finding of gonadotropin stability is unsurprising rather than unexplained. The present data do not permit localization of any underlying endocrine mechanism, because no dynamic hypothalamic–pituitary measure was obtained.

The co-occurrence of favorable changes across body composition, metabolic markers, several hormones, and multiple semen parameters raises the possibility that these domains are physiologically interconnected. Pathways such as reduced adiposity, improved insulin sensitivity, and altered androgen regulation have been proposed in the literature; however, the present study was not designed to test mechanisms, and the concurrent changes observed here cannot establish that metabolic or hormonal improvements caused the semen changes. Oxidative stress and inflammation are commonly proposed mechanistic links between exercise, metabolic change, and semen quality, but neither was measured in this study, so this pathway can be offered only as a plausible explanation drawn from the general literature, not as a demonstrated mechanism here. No mediation or path analysis was conducted, and the present data do not establish that any single domain caused or mediated change in another; these outcomes should therefore be understood as concurrently measured, interconnected findings rather than a validated causal sequence.

These findings should be interpreted in light of the study’s limited sample size, the non-randomized allocation of participants, multiple outcome testing, and the use of a single semen sample at each assessment point. The practical implications, methodological strengths and limitations, and recommendations for future research are presented separately below.

### 4.1. Practical Applications

The present findings suggest that structured combined aerobic and resistance exercise may represent a supportive lifestyle strategy for body composition and glucose regulation in healthy adult men. The concurrent changes observed in selected semen parameters and reproductive hormones also suggest that combined exercise may be associated with aspects of male reproductive physiology. However, these reproductive findings remain exploratory and should not be interpreted as evidence that exercise improves fertility, conception, pregnancy, or live-birth outcomes, none of which were assessed in the present study.

The findings do not support the use of exercise as a stand-alone treatment for male infertility. Practical interpretation should also consider the modest absolute magnitude of change in some outcomes, natural within-person variability in semen parameters, and the limited sample size. The results apply specifically to the healthy adult men included in this study and should not be directly generalized to infertile men, athletes, or individuals with obesity or metabolic disease. Accordingly, combined exercise may be considered a general health-promoting behavior with potential relevance to reproductive physiology rather than a proven reproductive therapy.

### 4.2. Strengths and Limitations

The principal strengths of this study include the use of a concurrent control group assessed at the same two time points as the exercise group and the simultaneous evaluation of body composition, glucose metabolism, reproductive hormones, and a broad panel of conventional and calculated semen indices within a single research framework. Sexual abstinence was standardized at three days before semen collection at both measurement points. Both statistically significant and non-significant findings were reported and interpreted, and effect sizes were provided alongside significance tests. The inclusion of total sperm count, total motile sperm count, and progressive motile sperm count also provided integrated summaries of sperm quantity and motility, although these indices were mathematically derived from the conventional semen parameters and should not be interpreted as independent confirmation of separate biological effects.

Several limitations should also be acknowledged. The sample was relatively small, with 17 participants in each group, which limits statistical precision and the generalizability of the results. No random-allocation procedure was used; therefore, selection or allocation bias cannot be excluded. Participants who chose to undertake supervised training may have been more motivated and health-conscious than controls and may also have made concurrent, unmeasured changes in diet, sleep, alcohol intake, or sexual behavior during the study. Such co-interventions would bias the comparison in favor of the exercise group, so the observed between-group differences may overestimate any true effect of exercise. Baseline comparisons and baseline-adjusted analyses can account only for measured baseline differences, not for unmeasured characteristics or co-interventions. Twenty outcome variables were analyzed using separate statistical models and the primary analysis was not corrected for multiple comparisons, increasing the risk of type I error. In the Holm–Bonferroni sensitivity analysis, the semen volume, estradiol, and total sperm count interactions were no longer statistically significant; these findings, and any finding with a *p*-value close to the conventional threshold, therefore require cautious interpretation and independent replication. Diagnostic checks further indicated non-normal or heteroscedastic change scores for several outcomes, violation of the homogeneity-of-regression-slopes assumption in the ANCOVA models for nine outcomes, and a fat-free-mass result that depended on a single influential observation. Kruger strict morphology also showed almost no within-participant change, which made the related diagnostics numerically unstable (Supplementary Tables S1–S4).

One control-group value was missing for fasting insulin and HOMA-IR and was handled through complete-case analysis. In addition, only one semen sample was collected at each assessment point. Semen parameters may vary within individuals because of biological fluctuation, sample completeness, illness, stress, sleep, medication or supplement use, and laboratory measurement variability. Consequently, regression toward the mean and natural variability cannot be excluded as possible contributors to some of the observed changes. The WHO laboratory manual recommends examining more than one sample when characterizing an individual’s semen quality [40]; the use of a single sample per time point is therefore a material limitation for interpreting change in semen parameters.

Comprehensive monitoring of diet, sleep, psychological stress, medication use, supplement use, and habitual physical activity, including activity in the control group, was not documented. Some aspects of the exercise dose and laboratory procedures were also reported with limited detail, restricting reproducibility and precise comparison with other interventions. In particular, session attendance and adherence to the prescribed exercise intensity were not systematically recorded, so the exercise dose actually delivered could not be quantified. Free or calculated free testosterone, albumin, the free androgen index, sperm DNA fragmentation, seminal leukocytes, sperm agglutination, oxidative-stress markers, and inflammatory biomarkers were not assessed. No mediation or path analysis was performed, and the study did not evaluate conception, pregnancy, or live-birth outcomes. Accordingly, the observed semen and hormonal changes cannot be interpreted as evidence of improved fertility or clinical reproductive benefit. Finally, several partial eta-squared values were very large, but their precision may have been influenced by the small sample, multiple outcome testing, sampling variability, and the non-randomized study design. These effect sizes should therefore not be equated with large clinical benefits. In small samples, statistically significant effect sizes also tend to be overestimated, and the 95% confidence intervals reported in Section 3 should be considered when judging the plausible magnitude of the between-group differences.

### 4.3. Recommendations for Future Research

Future studies should use larger, adequately powered samples and clearly documented random-allocation and allocation-concealment procedures. Longer intervention periods and extended follow-up would help determine whether outcomes that remained unchanged, including sperm morphology, respond differently over time. Repeated semen sampling at baseline and after the intervention would reduce the influence of natural within-person variability and provide more reliable estimates of semen-quality change.

Exercise interventions should be reported in sufficient detail to permit replication, including training frequency, intensity, duration, mode, progression, supervision, and adherence. Future protocols should also monitor habitual physical activity, diet, sleep, psychological stress, medication use, and supplement use in both intervention and control groups.

Mechanistic investigations should include free or calculated free testosterone, albumin, sperm DNA fragmentation, oxidative-stress markers, and inflammatory biomarkers. Repeated hormonal measurements may also be valuable for hormones influenced by circadian or pulsatile variation. Studies examining multiple outcomes should prospectively define primary and secondary outcomes, establish an explicit strategy for multiple-comparison correction, and report confidence intervals alongside effect estimates. Sensitivity analyses should be considered for semen variables with skewed distributions. In adequately powered samples, mediation or path analysis could help determine whether metabolic or hormonal changes statistically account for changes in semen parameters. Research intended to establish clinical fertility relevance may additionally consider conception, pregnancy, or live-birth outcomes.

### 4.4. Conclusions

In healthy men, a 12-week combined exercise program was associated with favorable changes in body composition, glucose metabolism, most hormonal measures, and several conventional and calculated semen parameters, while FSH, LH, non-progressive motility, and morphology remained unchanged. These findings are best regarded as a preliminary, exploratory signal of an association between structured combined exercise and multiple interconnected physiological domains in healthy men, rather than confirmed evidence of a reproductive or metabolic benefit. The small sample, non-randomized study design, absence of a multiplicity correction, single-timepoint semen sampling, and lack of oxidative stress, inflammatory, and free-androgen measures all constrain the strength of these conclusions. Larger, randomized, longer-duration studies incorporating these missing measurements are needed before any clinical or reproductive-health recommendation can be drawn.

## Figures and Tables

**Table 1:** Changes in body composition following the 12-week intervention.

Variable	Group	Baseline Mean ± SD	Week 12 Mean ± SD	Mean Change	Time × Group F(1, 32)
BMI, kg/m^2^	Exercise	22.19 ± 1.41	21.62 ± 1.34	−0.57	282.726, *p* < 0.001, η*p*^2^ = 0.898
	Control	22.45 ± 1.27	22.46 ± 1.23	+0.01	
Body fat, %	Exercise	15.10 ± 3.05	13.78 ± 2.71	−1.32	167.586, *p* < 0.001, η*p*^2^ = 0.840
	Control	15.56 ± 2.71	15.64 ± 2.67	+0.08	
Body mass, kg	Exercise	70.44 ± 4.94	68.62 ± 4.70	−1.81	327.334, *p* < 0.001, η*p*^2^ = 0.911
	Control	71.57 ± 4.77	71.62 ± 4.78	+0.04	
Fat-free mass, kg	Exercise	59.42 ± 4.15	59.12 ± 3.64	−0.30	0.849, *p* = 0.364, η*p*^2^ = 0.026
	Control	60.33 ± 2.80	60.35 ± 2.83	+0.02	

Note: Values are presented as mean ± standard deviation. BMI: body mass index; η*p*^2^: partial eta squared.

**Table 2:** Changes in glucose-metabolism markers following the 12-week intervention.

Variable	Group	Baseline Mean ± SD	Week 12 Mean ± SD	Mean Change	Time × Group
Fasting glucose, mg/dL	Exercise	90.71 ± 5.44	87.44 ± 5.13	−3.27	F(1, 32), 107.397, *p* < 0.001, η*p*^2^ = 0.770
	Control	89.06 ± 4.15	89.27 ± 4.58	+0.21	
Fasting insulin, µIU/mL	Exercise	8.18 ± 4.00	6.80 ± 3.25	−1.38	F(1, 31), 39.631, *p* < 0.001, η*p*^2^ = 0.561
	Control	7.27 ± 3.51	7.21 ± 3.37	−0.06	
HOMA-IR	Exercise	1.85 ± 0.95	1.48 ± 0.74	−0.37	F(1, 31), 43.205, *p* < 0.001, η*p*^2^ = 0.582
	Control	1.60 ± 0.81	1.60 ± 0.79	−0.01	

Note: Values are presented as mean ± standard deviation. HOMA-IR: homeostatic model assessment of insulin resistance; η*p*^2^: partial eta squared. Due to one missing control-group value, fasting insulin and HOMA-IR analyses were performed with 17 participants in the exercise group and 16 participants in the control group.

**Table 3:** Changes in hormonal profile following the 12-week intervention.

Variable	Group	Baseline Mean ± SD	Week 12 Mean ± SD	Mean Change	Time × Group F(1, 32)
Total testosterone, ng/dL	Exercise	624.46 ± 72.51	681.93 ± 83.40	+57.46	203.808, *p* < 0.001, η*p*^2^ = 0.864
	Control	627.71 ± 48.62	627.42 ± 52.18	−0.29	
FSH, IU/L	Exercise	4.97 ± 0.55	4.98 ± 0.63	+0.01	0.808, *p* = 0.376, η*p*^2^ = 0.025
	Control	5.11 ± 1.04	5.18 ± 1.11	+0.07	
LH, IU/L	Exercise	4.02 ± 0.72	3.94 ± 0.75	−0.07	0.056, *p* = 0.815, η*p*^2^ = 0.002
	Control	3.79 ± 0.45	3.73 ± 0.45	−0.06	
SHBG, nmol/L	Exercise	34.33 ± 3.08	35.98 ± 3.22	+1.66	19.131, *p* < 0.001, η*p*^2^ = 0.374
	Control	34.53 ± 3.69	34.53 ± 3.10	0.00	
Estradiol, pg/mL	Exercise	25.03 ± 2.33	24.08 ± 2.49	−0.95	5.508, *p* = 0.025, η*p*^2^ = 0.147
	Control	25.58 ± 2.61	25.31 ± 2.61	−0.26	
Prolactin, ng/mL	Exercise	9.59 ± 1.40	9.08 ± 1.43	−0.52	17.887, *p* < 0.001, η*p*^2^ = 0.359
	Control	9.14 ± 1.12	9.21 ± 1.25	+0.07	

Note: Values are presented as mean ± standard deviation. FSH: follicle-stimulating hormone; LH: luteinizing hormone; SHBG: sex hormone-binding globulin; η*p*^2^: partial eta squared.

**Table 4:** Changes in conventional semen parameters and predefined derived semen indices.

Variable	Group	Baseline Mean ± SD	Week 12 Mean ± SD	Mean Change	Time × Group F(1, 32)
Semen volume, mL	Exercise	2.10 ± 1.22	2.16 ± 1.22	+0.06	5.241, *p* = 0.029, η*p*^2^ = 0.141
	Control	1.58 ± 1.36	1.58 ± 1.36	+0.00	
Sperm concentration, million/mL	Exercise	22.88 ± 15.72	25.57 ± 17.18	+2.69	46.928, *p* < 0.001, η*p*^2^ = 0.595
	Control	25.24 ± 28.86	25.85 ± 30.62	+0.61	
Total motility (PR + NP), %	Exercise	40.53 ± 12.24	44.52 ± 11.74	+3.99	112.707, *p* < 0.001, η*p*^2^ = 0.779
	Control	40.88 ± 16.83	40.02 ± 16.72	−0.86	
Progressive motility, %	Exercise	34.94 ± 12.78	38.46 ± 12.13	+3.52	107.672, *p* < 0.001, η*p*^2^ = 0.771
	Control	33.06 ± 14.22	32.03 ± 14.26	−1.03	
Non-progressive motility, %	Exercise	5.59 ± 1.66	6.05 ± 1.76	+0.46	0.762, *p* = 0.389, η*p*^2^ = 0.023
	Control	8.12 ± 5.94	7.99 ± 5.69	−0.12	
Kruger strict morphology, %	Exercise	5.29 ± 1.10	5.35 ± 1.00	+0.06	1.000, *p* = 0.325, η*p*^2^ = 0.030
	Control	4.59 ± 1.62	4.59 ± 1.62	0.00	
Total sperm count, million	Exercise	50.56 ± 59.16	58.38 ± 66.59	+7.82	7.106, *p*= 0.012, η*p*^2^ = 0.182
	Control	47.79 ± 64.72	49.42 ± 68.11	+1.63	
Total motile sperm count, million	Exercise	19.68 ± 23.65	25.56 ± 30.36	+5.88	11.112, *p* = 0.002, η*p*^2^ = 0.258
	Control	16.80 ± 25.04	17.08 ± 25.74	+0.28	
Progressive motile sperm count, million	Exercise	17.02 ± 20.80	21.79 ± 25.36	+4.77	16.413, *p* < 0.001, η*p*^2^ = 0.339
	Control	14.49 ± 22.01	14.62 ± 22.63	+0.13	

Note: Values are presented as mean ± standard deviation. η*p*^2^: partial eta squared. Sperm concentration was analyzed using log10(x + 1)-transformed values, while descriptive values are presented in original units.

## Data Availability

The raw data supporting the conclusions of this article will be made available by the authors, without undue reservation.

## Supplementary Materials

The following supporting information can be downloaded at https://www.mdpi.com/article/10.3390/jcm15218415/s1. Table S1: Between-group sensitivity analyses of change scores; Table S2: Baseline-adjusted ANCOVA results; Table S3: Assumption checks for change-score comparisons; Table S4: ANCOVA assumption and influence diagnostics.
