# Supplementary material for: Semen Quality and Metabolic Biomarker Changes Following a 12-Week Combined Exercise Program in Healthy Adult Men: A Non-Randomized Controlled Intervention Study
Source: J Clin Med. 2026 Oct 29;15(21):8415. doi: 10.3390/jcm15218415 (PMC13764019; doi:10.3390/jcm15218415)
Supplement: Supplementary file 1 [file jcm-15-08415-s001.zip › jcm-4552435-supplementary.pdf]

Supplementary Statistical Sensitivity Analyses

The analyses below were calculated from the participant-level study dataset. The exercise group included 17 participants and the control group included 17 participants; fasting insulin and HOMA-IR analyses used 17 exercise and 16 control participants because one control observation was missing.

Change scores were defined as week 12 minus baseline. Sperm concentration was analyzed using log10(x+1)-transformed values. No observations were removed on the basis of diagnostic flags.

Supplementary Table S1. Between-group sensitivity analyses of change scores.

Outcome (unit / scale)	n E/C	Δ Exercise Mean ± SD	Δ Control Mean ± SD	Mean difference (95% CI)	Welch t (df)	Welch p	Welch q (BH)	MW U	MW p	MW q (BH)
BMI (kg/m²)	17/17	−0.571 ± 0.105	0.010 ± 0.097	−0.581 (−0.651, −0.510)	−16.81 (31.79)	2.23e-17	2.45e-16	0.0	6.47e-07	2.59e-06
Body fat percentage (%)	17/17	−1.324 ± 0.377	0.076 ± 0.239	−1.400 (−1.622, −1.178)	−12.95 (27.06)	4.16e-13	2.29e-12	0.0	6.70e-07	2.59e-06
Body mass (kg)	17/17	−1.812 ± 0.296	0.044 ± 0.303	−1.856 (−2.065, −1.647)	−18.09 (31.98)	2.32e-18	5.10e-17	0.0	6.70e-07	2.59e-06
Fat-free mass (kg)	17/17	−0.303 ± 1.423	0.016 ± 0.132	−0.319 (−1.053, 0.414)	−0.92 (16.28)	0.370	0.410	17.0	1.22e-05	2.23e-05
Fasting glucose (mg/dL)	17/17	−3.271 ± 0.486	0.212 ± 1.298	−3.482 (−4.182, −2.782)	−10.36 (20.40)	1.41e-09	4.42e-09	0.0	6.86e-07	2.59e-06
Fasting insulin (μIU/mL)	17/16	−1.379 ± 0.770	−0.057 ± 0.344	−1.322 (−1.748, −0.896)	−6.43 (22.41)	1.66e-06	3.90e-06	5.0	2.59e-06	5.70e-06
HOMA-IR	17/16	−0.368 ± 0.211	−0.006 ± 0.066	−0.362 (−0.475, −0.250)	−6.74 (19.29)	1.77e-06	3.90e-06	1.0	1.26e-06	3.09e-06
Total testosterone (ng/dL)	17/17	57.465 ± 14.073	−0.294 ± 8.956	57.759 (49.459, 66.058)	14.28 (27.13)	3.89e-14	2.85e-13	289.0	7.05e-07	2.59e-06
FSH (IU/L)	17/17	0.009 ± 0.210	0.071 ± 0.191	−0.062 (−0.202, 0.078)	−0.90 (31.72)	0.376	0.410	116.0	0.335	0.363
LH (IU/L)	17/17	−0.075 ± 0.208	−0.058 ± 0.213	−0.017 (−0.164, 0.130)	−0.24 (31.98)	0.815	0.815	141.5	0.931	0.931
SHBG (nmol/L)	17/17	1.656 ± 0.993	−0.002 ± 1.207	1.658 (0.885, 2.432)	4.37 (30.86)	1.29e-04	2.58e-04	250.0	2.99e-04	4.10e-04
Estradiol (pg/mL)	17/17	−0.955 ± 0.775	−0.265 ± 0.932	−0.690 (−1.290, −0.090)	−2.35 (30.97)	0.026	0.035	81.0	0.030	0.039
Prolactin (ng/mL)	17/17	−0.517 ± 0.395	0.072 ± 0.416	−0.589 (−0.872, −0.305)	−4.23 (31.92)	1.84e-04	3.37e-04	34.0	1.51e-04	2.22e-04
Semen volume (mL)	17/17	0.059 ± 0.028	0.004 ± 0.097	0.056 (0.005, 0.107)	2.29 (18.75)	0.034	0.044	188.0	0.138	0.169
Sperm concentration [log10(x+1)] (log10(million/mL + 1))	17/17	0.053 ± 0.020	0.003 ± 0.023	0.051 (0.036, 0.066)	6.85 (31.17)	1.09e-07	2.99e-07	287.0	9.86e-07	2.71e-06
Total motility (%)	17/17	3.988 ± 0.941	−0.859 ± 1.631	4.847 (3.908, 5.786)	10.62 (25.59)	7.24e-11	2.65e-10	287.5	8.95e-07	2.71e-06
Progressive motility (%)	17/17	3.524 ± 0.990	−1.029 ± 1.514	4.553 (3.654, 5.452)	10.38 (27.57)	5.04e-11	2.22e-10	289.0	6.93e-07	2.59e-06
Non-progressive motility (%)	17/17	0.459 ± 1.182	−0.124 ± 2.484	0.582 (−0.798, 1.963)	0.87 (22.89)	0.392	0.410	180.5	0.220	0.254
Kruger strict morphology (%)	17/17	0.059 ± 0.243	0.000 ± 0.000	0.059 (−0.066, 0.184)	1.00 (16.00)	0.332	0.406	153.0	0.347	0.363
Total sperm count (million)	17/17	7.818 ± 7.597	1.634 ± 5.812	6.184 (1.446, 10.922)	2.67 (29.95)	0.012	0.018	256.0	1.31e-04	2.06e-04
Total motile sperm count (million)	17/17	5.877 ± 6.717	0.283 ± 1.661	5.594 (2.068, 9.121)	3.33 (17.95)	0.004	0.006	266.0	3.06e-05	5.18e-05
Progressive motile sperm count (million)	17/17	4.766 ± 4.573	0.128 ± 1.169	4.638 (2.234, 7.043)	4.05 (18.08)	7.43e-04	0.001	280.0	3.30e-06	6.61e-06

Note. Δ = week 12 − baseline; the mean difference is Δ(exercise) − Δ(control), with a Welch 95% confidence interval. Welch's t-test does not assume equal group variances. Mann–Whitney U tests compare change-score distributions and are not necessarily tests of medians unless distribution shapes are similar. q values are Benjamini–Hochberg adjusted across the 22 outcomes separately within each test family. Sperm concentration is on the log10(x+1) scale.

Supplementary Table S2. Baseline-adjusted ANCOVA results.

Outcome (unit / scale)	N	Adjusted group coeffi cient, E–C (HC3 95% CI)	p (HC3)	q (BH, HC3)	p (conventional)	Partial η² (conventional)	Group × baseline p
BMI (kg/m²)	34	−0.592 (−0.653, −0.530)	4.84e-19	1.06e-17	1.28e-19	0.932	0.479
Body fat percentage (%)	34	−1.433 (−1.614, −1.252)	1.22e-16	6.74e-16	1.97e-17	0.905	5.15e-04
Body mass (kg)	34	−1.884 (−2.087, −1.682)	1.26e-18	1.38e-17	4.61e-19	0.926	0.016
Fat-free mass (kg)	34	−0.426 (−1.006, 0.155)	0.145	0.177	0.199	0.053	0.068
Fasting glucose (mg/dL)	34	−3.457 (−4.170, −2.744)	4.21e-11	1.32e-10	3.19e-11	0.763	0.108
Fasting insulin (µIU/mL)	33	−1.204 (−1.489, −0.920)	1.21e-09	3.33e-09	1.90e-10	0.746	2.12e-06
HOMA-IR	33	−0.328 (−0.411, −0.244)	6.23e-09	1.52e-08	2.75e-10	0.740	2.82e-09
Total testosterone (ng/dL)	34	58.138 (51.112, 65.164)	3.54e-17	2.60e-16	7.16e-18	0.911	0.157
FSH (IU/L)	34	−0.053 (−0.196, 0.090)	0.457	0.503	0.441	0.019	0.777
LH (IU/L)	34	−0.010 (−0.163, 0.142)	0.890	0.890	0.890	0.001	0.466
SHBG (nmol/L)	34	1.634 (0.873, 2.394)	1.25e-04	2.29e-04	7.58e-05	0.401	0.081
Estradiol (pg/mL)	34	−0.707 (−1.360, −0.053)	0.035	0.045	0.025	0.152	0.529
Prolactin (ng/mL)	34	−0.592 (−0.899, −0.284)	4.54e-04	7.69e-04	2.66e-04	0.353	0.545
Semen volume (mL)	34	0.058 (0.005, 0.112)	0.034	0.045	0.029	0.145	0.969
Sperm concentration [log10(x+1)] (log10(million/mL + 1))	34	0.052 (0.035, 0.068)	5.90e-07	1.30e-06	1.24e-07	0.600	0.003
Total motility (%)	34	4.839 (3.863, 5.816)	2.49e-11	9.14e-11	5.41e-12	0.789	0.348
Progressive motility (%)	34	4.600 (3.682, 5.519)	1.93e-11	8.48e-11	6.63e-12	0.786	0.131
Non-progressive motility (%)	34	0.246 (−1.265, 1.756)	0.742	0.778	0.719	0.004	0.826
Kruger strict morphology (%)	34	0.085 (−0.096, 0.266)	0.346	0.401	0.159	0.063	0.007
Total sperm count (million)	34	5.951 (2.187, 9.715)	0.003	0.004	4.05e-04	0.336	0.001
Total motile sperm count (million)	34	5.170 (2.139, 8.201)	0.002	0.002	1.02e-04	0.390	8.12e-16
Progressive motile sperm count (million)	34	4.340 (2.418, 6.261)	6.62e-05	1.32e-04	2.98e-06	0.511	2.17e-15

Note. ANCOVA model: week-12 outcome = group + baseline outcome. The adjusted coefficient is exercise minus control. HC3 heteroscedasticity-robust confidence intervals and p-values are provided alongside conventional OLS p-values and partial η². q values are Benjamini–Hochberg adjusted across the 22 HC3 ANCOVA tests. A group × baseline interaction p<.05 indicates evidence against homogeneity of regression slopes; for those outcomes, the simple ANCOVA coefficient is a screening estimate and should not be interpreted as a definitive adjusted effect.

Supplementary Table S3. Assumption checks for change-score comparisons.

Outcome (unit / scale)	n E/C	Shapiro–Wilk Exercise W (p)	Shapiro–Wilk Control W (p)	Levene test F (p)	Max ∣externally studentized residual∣	Max Cook's D	Screening flags
BMI (kg/m²)	17/17	0.918 (p=0.138)	0.912 (p=0.106)	F=0.02; p=0.884	2.54	0.172	Cook's D>4/N
Body fat percentage (%)	17/17	0.922 (p=0.158)	0.916 (p=0.124)	F=1.56; p=0.221	2.36	0.153	Cook's D>4/N
Body mass (kg)	17/17	0.921 (p=0.153)	0.915 (p=0.120)	F=0.00; p=0.959	2.14	0.128	Cook's D>4/N
Fat-free mass (kg)	17/17	0.379 (p=1.43e-07)	0.951 (p=0.465)	F=1.12; p=0.298	33.78	0.974	Shapiro–Wilk p<.05 in at least one group; Studentized-residual flag; Cook's D>4/N
Fasting glucose (mg/dL)	17/17	0.938 (p=0.292)	0.937 (p=0.289)	F=22.96; p=3.64e-05	2.51	0.169	Levene p<.05; Cook's D>4/N
Fasting insulin (µIU/mL)	17/16	0.867 (p=0.020)	0.962 (p=0.698)	F=3.11; p=0.087	4.41	0.381	Shapiro–Wilk p<.05 in at least one group; Studentized-residual flag; Cook's D>4/N
HOMA-IR	17/16	0.883 (p=0.035)	0.940 (p=0.348)	F=5.18; p=0.030	4.35	0.375	Shapiro–Wilk p<.05 in at least one group; Levene p<.05; Studentized-residual flag; Cook's D>4/N
Total testosterone (ng/dL)	17/17	0.978 (p=0.940)	0.950 (p=0.451)	F=3.31; p=0.078	2.67	0.187	Cook's D>4/N
FSH (IU/L)	17/17	0.919 (p=0.145)	0.973 (p=0.865)	F=0.44; p=0.513	1.99	0.114	No flag at stated screening thresholds
LH (IU/L)	17/17	0.945 (p=0.381)	0.985 (p=0.989)	F=0.07; p=0.789	2.52	0.170	Cook's D>4/N
SHBG (nmol/L)	17/17	0.974 (p=0.891)	0.985 (p=0.990)	F=0.17; p=0.687	2.54	0.172	Cook's D>4/N
Estradiol (pg/mL)	17/17	0.934 (p=0.251)	0.902 (p=0.072)	F=0.19; p=0.669	3.79	0.316	Studentized-residual flag; Cook's D>4/N
Prolactin (ng/mL)	17/17	0.879 (p=0.031)	0.956 (p=0.567)	F=0.04; p=0.838	2.62	0.181	Shapiro–Wilk p<.05 in at least one group; Cook's D>4/N
Semen volume (mL)	17/17	0.902 (p=0.073)	0.907 (p=0.091)	F=25.32; p=1.81e-05	2.08	0.122	Levene p<.05; Cook's D>4/N
Sperm concentration [log10(x+1)] (log10(million/mL + 1))	17/17	0.813 (p=0.003)	0.891 (p=0.048)	F=0.90; p=0.350	2.00	0.114	Shapiro–Wilk p<.05 in at least one group
Total motility (%)	17/17	0.923 (p=0.169)	0.926 (p=0.186)	F=4.85; p=0.035	2.99	0.224	Levene p<.05; Cook's D>4/N
Progressive motility (%)	17/17	0.837 (p=0.007)	0.890 (p=0.046)	F=2.62; p=0.115	2.05	0.120	Shapiro–Wilk p<.05 in at least one group; Cook's D>4/N
Non-progressive motility (%)	17/17	0.723 (p=2.16e-04)	0.936 (p=0.273)	F=5.03; p=0.032	2.89	0.213	Shapiro–Wilk p<.05 in at least one group; Levene p<.05; Cook's D>4/N
Kruger strict morphology (%)	17/17	0.262 (p=2.17e-08)	Not testable (constant)	F=1.00; p=0.325	Not finite†	1.000	Shapiro–Wilk p<.05 in at least one group; Shapiro–Wilk not testable for constant changes; Studentized-residual flag; Cook's D>4/N
Total sperm count (million)	17/17	0.637 (p=2.41e-05)	0.655 (p=3.78e-05)	F=0.34; p=0.562	3.46	0.278	Shapiro–Wilk p<.05 in at least one group; Studentized-residual flag; Cook's D>4/N
Total motile sperm count (million)	17/17	0.576 (p=6.12e-06)	0.792 (p=0.002)	F=2.37; p=0.133	4.80	0.426	Shapiro–Wilk p<.05 in at least one group; Studentized-residual flag; Cook's D>4/N
Progressive motile sperm count (million)	17/17	0.638 (p=2.48e-05)	0.833 (p=0.006)	F=2.79; p=0.105	4.64	0.410	Shapiro–Wilk p<.05 in at least one group; Studentized-residual flag; Cook's D>4/N

Note. Shapiro–Wilk tests were applied to change scores separately by group. Levene's test used median centering. Studentized residuals and Cook's distance are from the change-score model (Δ ~ group). Cook's D > 4/N and ∣externally studentized residual∣ > 3 are screening flags only and do not justify automatic case deletion. † A non-finite studentized residual indicates a numerically unstable diagnostic, usually associated with near-zero residual variance.

Supplementary Table S4. ANCOVA assumption and influence diagnostics.

Outcome (unit / scale)	N	Group × baseline p	ANCOVA residual Shapiro–Wilk W (p)	Breusch–Pagan LM (p)	Max ∣externally studentized residual∣	Max Cook's D	Screening flags
BMI (kg/m²)	34	0.479	0.970 (p=0.450)	LM=0.55; p=0.760	1.99	0.169	Cook's D>4/N
Body fat percentage (%)	34	5.15e-04	0.956 (p=0.192)	LM=6.38; p=0.041	2.34	0.231	Slope homogeneity: group×baseline p<.05; Breusch–Pagan p<.05; Cook's D>4/N
Body mass (kg)	34	0.016	0.972 (p=0.514)	LM=7.49; p=0.024	2.09	0.129	Slope homogeneity: group×baseline p<.05; Breusch–Pagan p<.05; Cook's D>4/N
Fat-free mass (kg)	34	0.068	0.597 (p=1.78e-08)	LM=7.52; p=0.023	30.49	2.597	ANCOVA residual Shapiro–Wilk p<.05; Breusch–Pagan p<.05; Studentized-residual flag; Cook's D>4/N
Fasting glucose (mg/dL)	34	0.108	0.989 (p=0.976)	LM=13.14; p=0.001	2.60	0.167	Breusch–Pagan p<.05; Cook's D>4/N
Fasting insulin (μIU/mL)	33	2.12e-06	0.991 (p=0.991)	LM=9.69; p=0.008	3.08	0.826	Slope homogeneity: group×baseline p<.05; Breusch–Pagan p<.05; Studentized-residual flag; Cook's D>4/N
HOMA-IR	33	2.82e-09	0.985 (p=0.924)	LM=12.00; p=0.002	3.16	0.782	Slope homogeneity: group×baseline p<.05; Breusch–Pagan p<.05; Studentized-residual flag; Cook's D>4/N
Total testosterone (ng/dL)	34	0.157	0.976 (p=0.630)	LM=4.81; p=0.090	2.09	0.306	Cook's D>4/N
FSH (IU/L)	34	0.777	0.957 (p=0.193)	LM=0.67; p=0.716	1.96	0.149	Cook's D>4/N
LH (IU/L)	34	0.466	0.972 (p=0.513)	LM=0.00; p=0.999	2.44	0.136	Cook's D>4/N
SHBG (nmol/L)	34	0.081	0.979 (p=0.737)	LM=0.11; p=0.948	2.22	0.154	Cook's D>4/N
Estradiol (pg/mL)	34	0.529	0.962 (p=0.279)	LM=1.62; p=0.444	3.68	0.327	Studentized-residual flag; Cook's D>4/N
Prolactin (ng/mL)	34	0.545	0.970 (p=0.461)	LM=0.09; p=0.956	2.59	0.227	Cook's D>4/N
Semen volume (mL)	34	0.969	0.967 (p=0.393)	LM=14.46; p=7.25e-04	2.10	0.167	Breusch–Pagan p<.05; Cook's D>4/N
Sperm concentration [log10(x+1)] (log10(million/mL + 1))	34	0.003	0.936 (p=0.046)	LM=4.15; p=0.126	1.95	0.208	Slope homogeneity: group×baseline p<.05; ANCOVA residual Shapiro–Wilk p<.05; Cook's D>4/N
Total motility (%)	34	0.348	0.931 (p=0.034)	LM=5.69; p=0.058	3.16	0.572	ANCOVA residual Shapiro–Wilk p<.05; Studentized-residual flag; Cook's D>4/N
Progressive motility (%)	34	0.131	0.980 (p=0.787)	LM=9.73; p=0.008	2.34	0.188	Breusch–Pagan p<.05; Cook's D>4/N
Non-progressive motility (%)	34	0.826	0.952 (p=0.138)	LM=7.37; p=0.025	2.73	0.166	Breusch–Pagan p<.05; Cook's D>4/N
Kruger strict morphology (%)	34	0.007	0.471 (p=6.16e-10)	LM=4.41; p=0.110	>3†	1.741	Slope homogeneity: group×baseline p<.05; ANCOVA residual Shapiro–Wilk p<.05; Studentized-residual flag; Cook's D>4/N
Total sperm count (million)	34	0.001	0.805 (p=3.18e-05)	LM=13.19; p=0.001	7.51	2.304	Slope homogeneity: group×baseline p<.05; ANCOVA residual Shapiro–Wilk p<.05; Breusch–Pagan p<.05; Studentized-residual flag; Cook's D>4/N
Total motile sperm count (million)	34	8.12e-16	0.848 (p=2.59e-04)	LM=23.04; p=9.93e-06	5.48	1.981	Slope homogeneity: group×baseline p<.05; ANCOVA residual Shapiro–Wilk p<.05; Breusch–Pagan p<.05; Studentized-residual flag; Cook's D>4/N
Progressive motile sperm count (million)	34	2.17e-15	0.919 (p=0.015)	LM=25.36; p=3.11e-06	3.80	1.287	Slope homogeneity: group×baseline p<.05; ANCOVA residual Shapiro–Wilk p<.05; Breusch–Pagan p<.05; Studentized-residual flag; Cook's D>4/N

Note. The group × baseline interaction assesses homogeneity of regression slopes. Breusch–Pagan p<.05 suggests residual heteroscedasticity; Shapiro–Wilk p<.05 suggests departure from residual normality. Cook's D > 4/N and an externally studentized residual above 3 are screening flags only. No cases were removed. † The morphology ANCOVA diagnostic is numerically unstable because model residual variance is extremely small; this outcome should therefore be interpreted with caution.
